# Evaluation of risk stratification for acute kidney injury: a comparative analysis of EKFC, 2009 and 2021 CKD-EPI glomerular filtration estimating equations

**DOI:** 10.1007/s40620-023-01883-7

**Published:** 2024-02-12

**Authors:** Jeong Min Cho, Jung Hun Koh, Minsang Kim, Sehyun Jung, Semin Cho, Soojin Lee, Yaerim Kim, Yong Chul Kim, Hajeong Lee, Seung Seok Han, Kook-Hwan Oh, Kwon Wook Joo, Yon Su Kim, Dong Ki Kim, Sehoon Park

**Affiliations:** 1 Department of Internal Medicine, Seoul National University Hospital, 101 Daehak-Ro, Jongno-Gu, Seoul, 03080 South Korea; 2 Department of Internal Medicine, Gyeongsang National University College of Medicine, Jinju, South Korea; 3 Department of Internal Medicine, Chung-Ang University Gwangmyeong Hospital, Gwangmyeong, South Korea; 4 Department of Internal Medicine, Seoul National University College of Medicine, Seoul, South Korea; 5 Department of Internal Medicine, Uijeongbu Eulji University Medical Center, Uijeongbu, South Korea; 6 Department of Internal Medicine, Keimyung University School of Medicine, Daegu, South Korea

**Keywords:** Glomerular filtration rate, Kidney function tests, Creatinine, Acute kidney injury

## Abstract

**Background:**

The adoption of the 2021 CKD-EPIcr equation for glomerular filtration rate (GFR) estimation provided a race-free eGFR calculation. However, the discriminative performance for AKI risk has been rarely validated. We aimed to evaluate the differences in acute kidney injury (AKI) prediction or reclassification power according to the three eGFR equations.

**Methods:**

We performed a retrospective observational study within a tertiary hospital from 2011 to 2021. Acute kidney injury was defined according to KDIGO serum creatinine criteria. Glomerular filtration rate estimates were calculated by three GFR estimating equations: 2009 and 2021 CKD-EPIcr, and EKFC. In three equations, AKI prediction performance was evaluated with area under receiver operator curves (AUROC) and reclassification power was evaluated with net reclassification improvement analysis.

**Results:**

A total of 187,139 individuals, including 27,447 (14.7%) AKI and 159,692 (85.3%) controls, were enrolled. In the multivariable regression prediction model, the 2009 CKD-EPIcr model (continuous eGFR model 2, 0.7583 [0.755–0.7617]) showed superior performance in AKI prediction to the 2021 CKD-EPIcr (0.7564 [0.7531–0.7597], < 0.001) or EKFC model in AUROC (0.7577 [0.7543–0.761], < 0.001). Moreover, in reclassification of AKI, the 2021 CKD-EPIcr and EKFC models showed a worse classification performance than the 2009 CKD-EPIcr model. (− 7.24 [− 8.21–− 6.21], − 2.38 [− 2.72–− 1.97]).

**Conclusion:**

Regarding AKI risk stratification, the 2009 CKD-EPIcr equation showed better discriminative performance compared to the 2021 CKD-EPIcr equation in the study population.

**Graphical abstract:**

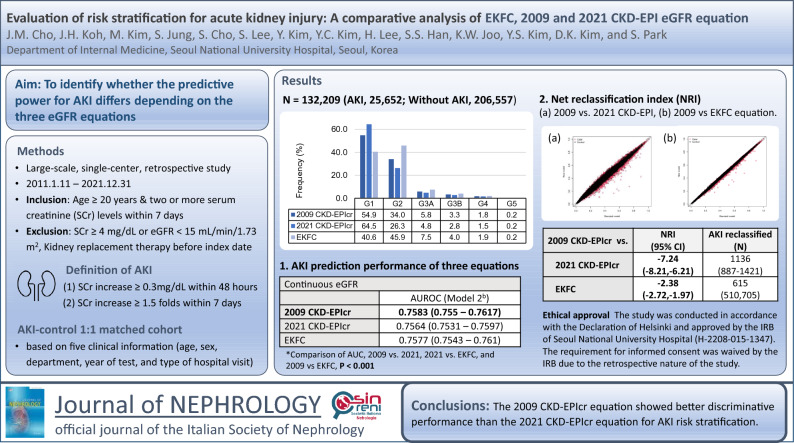

**Supplementary Information:**

The online version contains supplementary material available at 10.1007/s40620-023-01883-7.

## Introduction

Precise measurement of the glomerular filtration rate (GFR) is essential for the management of kidney diseases. The use of GFR thresholds encompasses various medical decisions, including diagnosis of chronic kidney disease (CKD), definition and risk stratification in acute kidney injury (AKI), initiation or discontinuation of medications, planning for kidney replacement therapy, and referral to nephrologists [1–3]. Because of the expensive, time-consuming, and impractical nature of directly measuring GFR through the clearance of exogenous filtration markers [4], estimated GFR (eGFR) calculated from serum creatinine and/or cystatin C is widely used in the clinical practice [5–7].

There are growing concerns regarding the use of race-based medicines and the recognition that the “race” variable reflects socio-cultural constructs rather than biological differences [8, 9]. The 2021 CKD Epidemiology Collaboration (CKD-EPI) equation, which excludes a race coefficient for the black population, was developed by the American Society of Nephrology and the National Kidney Foundation [10]. However, there are controversies surrounding the use of the 2021 CKD-EPI equation in estimating GFR, because of the lower accuracy in non-blacks and underestimation of the CKD prevalence due to overestimation of eGFR among high risk patients [11, 12].

Acute kidney injury is a significant health issue that causes substantial economic burden [13, 14] and poses an increased risk of short- and long-term morbidity and mortality [15, 16]. As AKI is a multifactorial syndrome, its treatment is often difficult even when early recognized and timely treated [14, 17, 18]. Therefore, the prediction of AKI development in high-risk patients and preemptive interventions for AKI might be critical for improving patient outcomes [19]. As a result, several studies for AKI risk assessment and prediction have been performed, and reduced baseline eGFR was found to be a core predictor of AKI [20, 21]. However, whether the predictive power for AKI differs depending on the eGFR equations chosen is as yet unknown.

In this study, we aimed to investigate whether there are differences in AKI prediction or reclassification according to different GFR-estimating equations in a cohort mostly comprising an Asian population. We hypothesized that the AKI prediction model based on the 2009 CKD-EPIcr equation would predict the development of AKI within 1 week from baseline better than the model based on the 2021 CKD-EPIcr or European Kidney Function Consortium (EKFC) equation [22]. The predictive models for AKI were developed from a large-scale retrospective observational cohort study conducted at a tertiary hospital.

## Methods

### Ethical considerations

The study was conducted in accordance with the Declaration of Helsinki and approved by the Institutional Review Board of Seoul National University Hospital (IRB No. H-2208-015-1347). The requirement for informed consent was waived by the IRB because of the retrospective observational nature of the study.

### Study design and participants

**Fig. 1:**
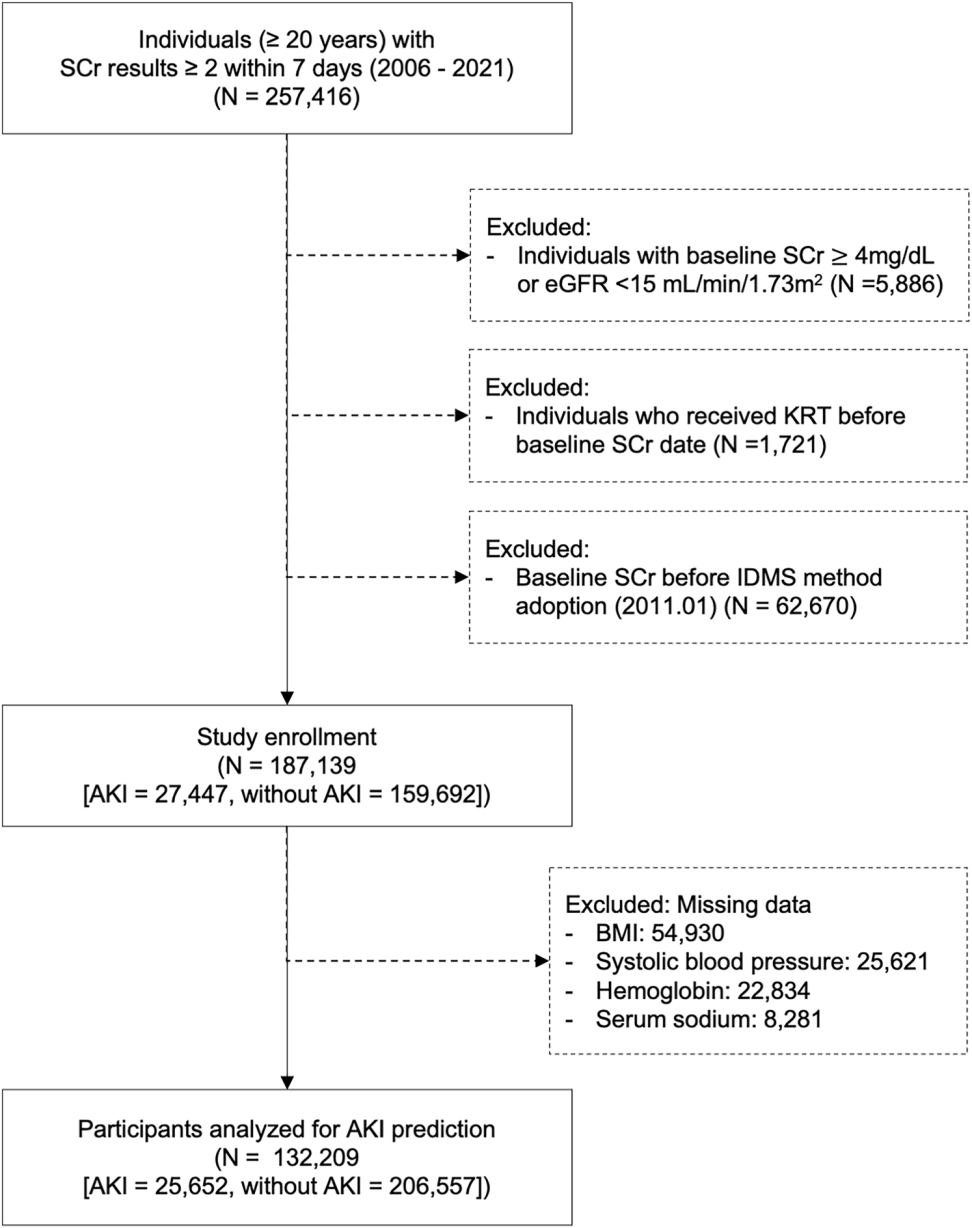
Study flow diagram. SCr, serum creatinine; eGFR, estimated glomerular filtration rate; AKI, acute kidney injury; KRT, kidney replacement therapy

This was a large-scale retrospective cohort study analyzing electronic health records generated between 2006 and 2021 at the Seoul National University Hospital, Seoul, Korea. A flowchart of the study is shown in Fig. 1. Individuals 20 years of age or older who underwent at least two serum creatinine (SCr) measurements within 7 days from January 1, 2006, to December 31, 2021, were screened for AKI. Among consecutive SCr results, the former was considered the baseline SCr level. In cases where the participants had more than three SCr results, the initial two SCr measurements were used to determine the AKI event. Acute kidney injury events were defined according to the Kidney Disease Improving Global Outcomes (KDIGO) SCr criteria. The exclusion criteria were as follows: (1) individuals with baseline SCr ≥ 4 mg/dL or eGFR < 15 mL/min/1.73 m^2^, (2) individuals who received kidney replacement therapy (i.e., hemodialysis, peritoneal dialysis, and continuous kidney replacement therapy) before baseline; and (3) individuals whose baseline SCr was measured before the adoption of isotope-dilution mass spectrometry (IDMS), which occurred on January 11, 2011. The remaining participants without missing data were included in the final cohort for construction of the AKI prediction model.

### Data collection and definition

Baseline characteristics (age, sex, body mass index (BMI), and systolic and diastolic blood pressure), laboratory results, and procedure (i.e., surgery or coronary artery intervention) data were collected at baseline. Hospital-related data, including the clinical department where the SCr test was conducted (internal medicine, surgical, or emergency medicine) and the type of hospital visit (admission, outpatient unit, or emergency room) were collected. Comorbidities, including hypertension, diabetes mellitus (DM), cardiovascular disease, and malignancy, were defined using the International Classification of Diseases, 10th revision (ICD-10) diagnostic codes, and relevant medical prescriptions. Prescription drugs included renin–angiotensin–aldosterone system blockers, beta-blockers, calcium channel blockers, diuretics, oral hypoglycemic agents, and insulin. Details of the ICD-10 and prescribed drug codes are presented in Supplemental Table 1. Among the participants who started kidney replacement therapy after an AKI event, the type and date of dialysis were collected. The medical records of the study participants were obtained either during admission, stay in the emergency room, or in the community setting.

Body mass index was categorized into < 18.5 (underweight), ≥ 18.5 and < 25 (reference range), ≥ 25 and < 30 (overweight), and ≥ 30 kg/m^2^ (obese). Systolic blood pressure was categorized into normal (≥ 100 and < 130 mmHg) and abnormal (< 100 or ≥ 130 mmHg). Anemia was defined as a hemoglobin level of < 12 g/dL for female and < 13 g/dL for male. Dysnatremia was defined as a serum sodium level < 135 or ≥ 145 mEq/dL.

### Three equations to estimate glomerular filtration rate

The eGFR was calculated using baseline SCr, sex, and age at baseline with three GFR estimating equations: 2009 CKD-EPIcr [7], 2021 CKD-EPIcr [10], and EKFC [22]. Based on each eGFR equation, participants were classified into the five categories of the KDIGO classification: ≥ 90, ≥ 60 and < 90, ≥ 45 and < 60, ≥ 30 and < 45, and ≥ 15 and < 30 mL/min/1.73 m^2^. Differences between the 2009 and 2021 CKD-EPIcr equations were calculated by subtracting the eGFR of the 2021 CKD-EPIcr from that of the 2009 CKD-EPIcr.

### Study outcome

The study outcome was the development of AKI within 1 week of baseline. Acute kidney injury was defined according to KDIGO SCr criteria as an increase in SCr by ≥ 0.3 mg/dL within 48 h or an increase in SCr to ≥ 1.5 times baseline within 7 days.

### Statistical analyses

Categorical variables were reported as numbers (percentages) and continuous variables as means (± SD) or medians [interquartile range (IQR)]. The bias of the 2009 and 2021 CKD-EPIcr equations was calculated by extracting the 2021 CKD-EPIcr from the 2009 CKD-EPIcr value and is presented in the histogram.

A multivariate regression prediction model was developed for the dataset. Model 1 was adjusted for age and sex, and Model 2 was adjusted for predictor variables, including demographic (age and sex) and clinical (BMI and systolic BP) values, laboratory values (presence of anemia, eGFR, and dysnatremia), clinical department (internal medicine, surgery, and emergency medicine), type of hospital visit (admission, outpatient unit, or emergency room), comorbidities (hypertension, DM, and malignancy), and procedures within 2 weeks before baseline. Two models were constructed based on the eGFR equations, wherein each model evaluated both continuous and categorical eGFR values.

The AKI prediction performance of the models was assessed by calculating the area under the receiver operator curve (AUROC) with 95% confidence interval (CI), and pairwise comparisons of AUROC were determined by the Delong method [23]. Analyses were repeated within subgroups of age, sex, DM, presence of CKD stage ≥ 3 (2009 CKD-EPIcr-estimated GFR threshold at 60 mL/min/1.73 m^2^), procedure (within 14 days before baseline), and year of baseline (2011–2013, 2014–2017, and 2018–2021).

To further assess reclassification, the net reclassification improvement analysis for AKI was implemented [24]. We set the 2009 CKD-EPIcr equation as a standard model, and the reclassification index of the 2021 CKD-EPIcr and EKFC equations was evaluated using continuous eGFR values. Bootstrapping with 100 replicates was used to construct 95% CIs for net reclassification improvement.

All statistical analyses were performed with R (version 4.2.3), and two-sided *P* values < 0.05 were considered statistically significant. The 1:1 exact matching was performed using “MatchIt” package in R. ROC and net reclassification improvement analyses were performed using the “pROC” and “nricens” package in R, respectively.

## Results

### Study population

Of 257,416 individuals who had at least two measurements of SCr within a week, participants whose baseline SCr was ≥ 4 mg/dL or eGFR < 15 mL/min/1.73 m^2^ (*N* = 5886), who had received kidney replacement therapy prior to baseline (*N* = 1721), or whose baseline SCr was measured after the adoption of IDMS (*N* = 62,670) were excluded. The study cohort consisted of 187,139 individuals, including participants who had at least one AKI event (*N* = 27,447) and those who did not experience any AKI event (*N* = 159,692). After excluding individuals with missing data (*N* = 54,930), 132,209 participants were analyzed.

### Baseline characteristics

The baseline characteristics of the study population are shown in Table 1. Of all the participants, 27,447 (14.7%) developed AKI, whereas 159,692 (85.3%) did not. The mean age of the overall participants was 58.2 ± 16.2 and 53.7% were male. In more than half of the study population (56.3%), the measurement of SCr level was conducted during hospitalization. In patients with AKI, comorbidities such as hypertension (42.9 vs. 29.7%), DM (38.0 vs. 13.1%), and malignancy (54.9 vs. 32.9%) were more prevalent compared to those without. In the AKI group, the mean hemoglobin (11.0 ± 2.2 g/dL), serum albumin (3.2 ± 0.7 mg/dL), and total CO_2_ (23.8 ± 4.5 mEq/L) were lower than those without AKI (12.9 ± 2.0 g/dL; 4.0 ± 0.6 mg/dL; and 25.3 ± 3.5 mEq/L, respectively). Dipstick albuminuria was more commonly observed in patients with AKI. The baseline SCr was similar between the two groups (AKI vs. without AKI group, 0.9 ± 0.4 vs. 0.9 ± 0.3 mg/dL). In the AKI group, 74.3% of patients were classified as AKI stage 1, while 10.5% and 15.2% were categorized as AKI stages 2 and 3, respectively. A total of 2656 (9.7%) patients with AKI received post-AKI kidney replacement therapy, including hemodialysis, peritoneal dialysis, or continuous renal replacement therapy.

**Table 1:** Study population

Variables	All participants	AKI	Without AKI
	(*N* = 187,139)	(*N* = 27,447)	(*N* = 159,692)
Age (year)	58.2 ± 16.2	62.4 ± 15.0	57.4 ± 16.3
Male [*n* (%)]	100,423 (53.7)	15,892 (57.9)	84,531 (52.9)
^a^Body mass index (kg/m^2^)	28.16 (24.78–31.98)	27.35 (23.84–31.24)	28.6 (25.3–32.4)
Systolic blood pressure (mmHg)	124.5 ± 20.4	123.1 ± 22.7	127.9 ± 22.0
Type of hospital visit [*n* (%)]			
Admission	105,405 (56.3)	21,132 (77.0)	84,273 (52.8)
Emergency room	32,407 (17.3)	3544 (12.9)	28,863 (18.1)
Outpatient clinic	49,327 (26.4)	2771 (10.1)	46,556 (29.2)
Department [*n* (%)]			
Internal medicine	63,851 (34.1)	11,777 (42.9)	52,074 (32.6)
Surgical	88,101 (47.1)	10,730 (39.1)	77,371 (48.5)
Emergency medicine	35,187 (18.8)	4940 (18.0)	30,247 (18.9)
Comorbidities [*n* (%)]			
Hypertension	62,174 (33.2)	14,724 (42.9)	47,450 (29.7)
Diabetes mellitus	31,401 (16.8)	10,444 (38.0)	20,957 (13.1)
Malignancy	67,610 (36.1)	15,058 (54.9)	52,552 (32.9)
Laboratory results			
Hemoglobin (g/dL)	12.4 ± 2.2	11.0 ± 2.2	12.9 ± 2.0
Albumin (g/dL)	3.7 ± 0.7	3.2 ± 0.7	4.0 ± 0.6
Total CO_2_ (mEq/L)	25.1 ± 3.7	23.8 ± 4.5	25.3 ± 3.5
Sodium (mEq/L)	139.1 ± 4.2	137.1 ± 5.8	139.7 ± 3.5
Calcium (mEq/L)	8.8 ± 0.7	8.4 ± 0.9	8.9 ± 0.6
Phosphorus (mEq/L)	3.4 ± 0.8	3.2 ± 0.9	3.4 ± 0.7
Total cholesterol (mg/dL)	161.8 ± 47.9	141.2 ± 54.9	170.6 ± 44.6
Glucose (mg/dL)	125.3 ± 53.3	136.0 ± 63.0	123.4 ± 51.0
^a^Dipstick urine albumin			
–	71,053 (59.7)	9701 (39.5)	61,352 (65.0)
Trace	26,576 (22.3)	5775 (23.5)	20,801 (22.0)
1 +–4 +	21,313 (18.0)	9054 (37.0)	12,259 (13.0)
Serum creatinine (SCr) (mg/dL)			
Baseline SCr	0.9 ± 0.4	0.9 ± 0.4	0.9 ± 0.3
SCr at AKI diagnosis	–	1.6 ± 3.1	–
Baseline eGFR (mL/min/1.73m^2^)			
2009 CKD-EPIcr	92.2 (77.7–104.0)	93.4 (66.9–111.2)	91.5 (78.2–102.5)
2021 CKD-EPIcr	96.5 (82.4–107.1)	97.5 (71.3–112.0)	96.0 (83.0–105.8)
EKFC	85.5 (72.0–98.6)	85.3 (62.4–102.8)	84.9 (72.4–97.2)
AKI stage [*n* (%)]			
1	–	20,387 (74.3)	–
2	–	2875 (10.5)	–
3	–	4184 (15.2)	–
Post-AKI KRT [*n* (%)]	–	2656 (9.7)	–

Continuous variables were presented as means ± SD or median (IQR) and categorical variables were presented as number (percentage)

The SCr levels at AKI diagnosis, AKI stage, and post-AKI KRT were calculated for the AKI group

*AKI* acute kidney injury, *eGFR* estimated glomerular filtration rate, *KRT* kidney replacement therapy

^*^Missing values: 54,930 (29.4%) for body mass index; 2,917 (10.6%) and 65,279 (40.9%) for dipstick urine albumin, AKI and without AKI groups, respectively

### Characteristics according to eGFR equations

**Fig. 2:**
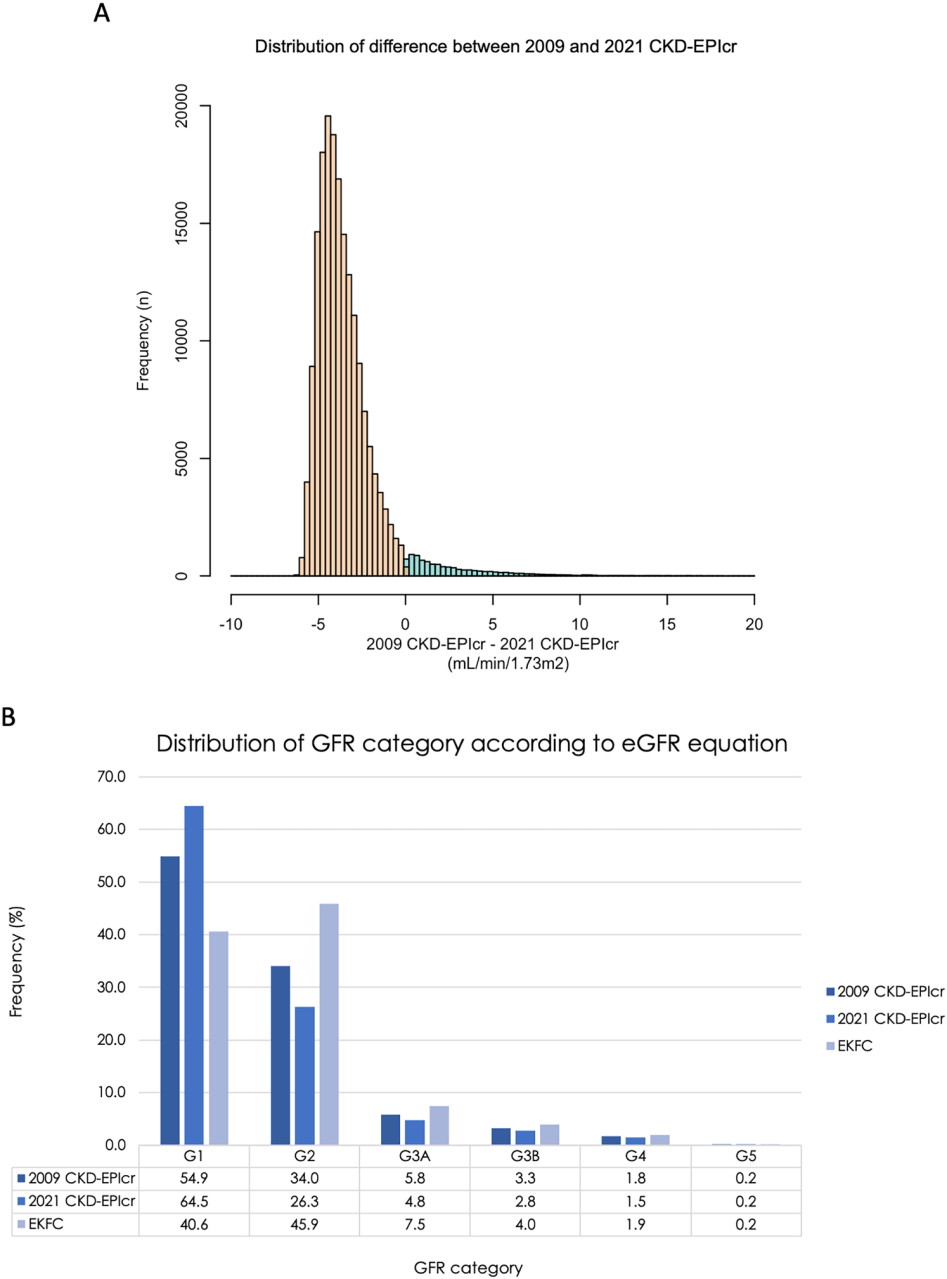
Distribution associated with eGFR equations. **A** The distribution of the difference between 2009 CKD-EPIcr and 2021 CKD-EPIcr was plotted in histogram. Orange-colored bars represent negative difference (95.0%), while skyblue-colored bars represent positive difference (5.0%). The median of negative and positive difference was was − 3.93 (− 4.63–− 2.99) and 1.95 (0.75–4.30) mL/min/1.73m^2^ respectively. **B** The distribution of the eGFR category according to each GFR estimating equation. The proportion of higher kidney function was the most observed in 2021 CKD-EPIcr equation than 2009 CKD-EPIcr or EKFC equation

The 2021 CKD-EPIcr equations (96.5 [82.4–107.1] mL/min/1.73 m^2^) exhibited a higher median baseline eGFR than that of the 2009 CKD-EPIcr (92.15 [77.73–104.02] mL/min/1.73 m^2^) or EKFC equation (85.50 [72.03–98.57] mL/min/1.73 m^2^) (Table 2). The distribution of the eGFR categories according to each eGFR equation is shown in Fig. 2A. Among the GFR estimating equations, the proportion of participants with eGFR ≥ 60 mL/min/1.73 m^2^ was highest in the 2021 CKD-EPIcr equation (90.8%) and smallest in the EKFC equation (86.4%). The proportion of patients with CKD stage ≥ 3 (eGFR < 60 mL/min/1.73 m^2^) was higher when calculated using either the EKFC (13.6%) or the 2009 CKD-EPIcr equation (11.1%) than when calculated using the 2021 CKD-EPIcr (9.2%) equation. In Fig. 2B, the differences between the 2009 and 2021 CKD-EPIcr equations are shown as a histogram. The proportion of participants with a negative difference (95.0%) exceeded the proportion of those with a positive difference (5.0%). The median difference was − 3.85 (− 4.60–− 2.79) mL/min/1.73 m^2^ in total. The median difference in the negative difference group was − 3.93 (− 4.63–− 2.99) mL/min/1.73 m^2^, while the median difference in the positive difference group was 1.95 (0.75–4.30) mL/min/1.73 m^2^.

**Table 2:** Distribution of baseline eGFR according to GFR estimating equation

	2009 CKD-EPIcr		2021 CKD-EPIcr		EKFC	
	Total	AKI	Total	AKI	Total	AKI
Baseline eGFR (mL/min/1.73m^2^)	92.2 (77.7–104.0)	93.4 (66.9–111.2)	96.5 (82.4–107.1)	97.5 (71.3–112.0)	85.5 (72.0–98.6)	85.3 (62.4–102.8)
eGFR category [*n* (%)]						
1	102,718 (54.9)	15,113 (55.1)	120,727 (64.5)	16,795 (61.2)	75,913 (40.6)	11,763 (42.9)
2	63,680 (34.0)	6644 (24.2)	49,170 (26.3)	5588 (20.4)	85,860 (45.9)	9322 (34.0)
3A	10,929 (5.8)	2202 (8.0)	8958 (4.8)	1975 (7.2)	13,962 (7.5)	2509 (9.1)
3B	6105 (3.3)	1814 (6.6)	5158 (2.8)	1643 (6.0)	7411 (4.0)	2092 (7.6)
4	3707 (2.0)	1674 (6.1)	3116 (1.7)	1446 (5.3)	3993 (0.2)	1761 (6.4)

*eGFR* estimated glomerular filtration rate, *AKI* acute kidney injury

### Performance comparison in AKI prediction

**Fig. 3:**
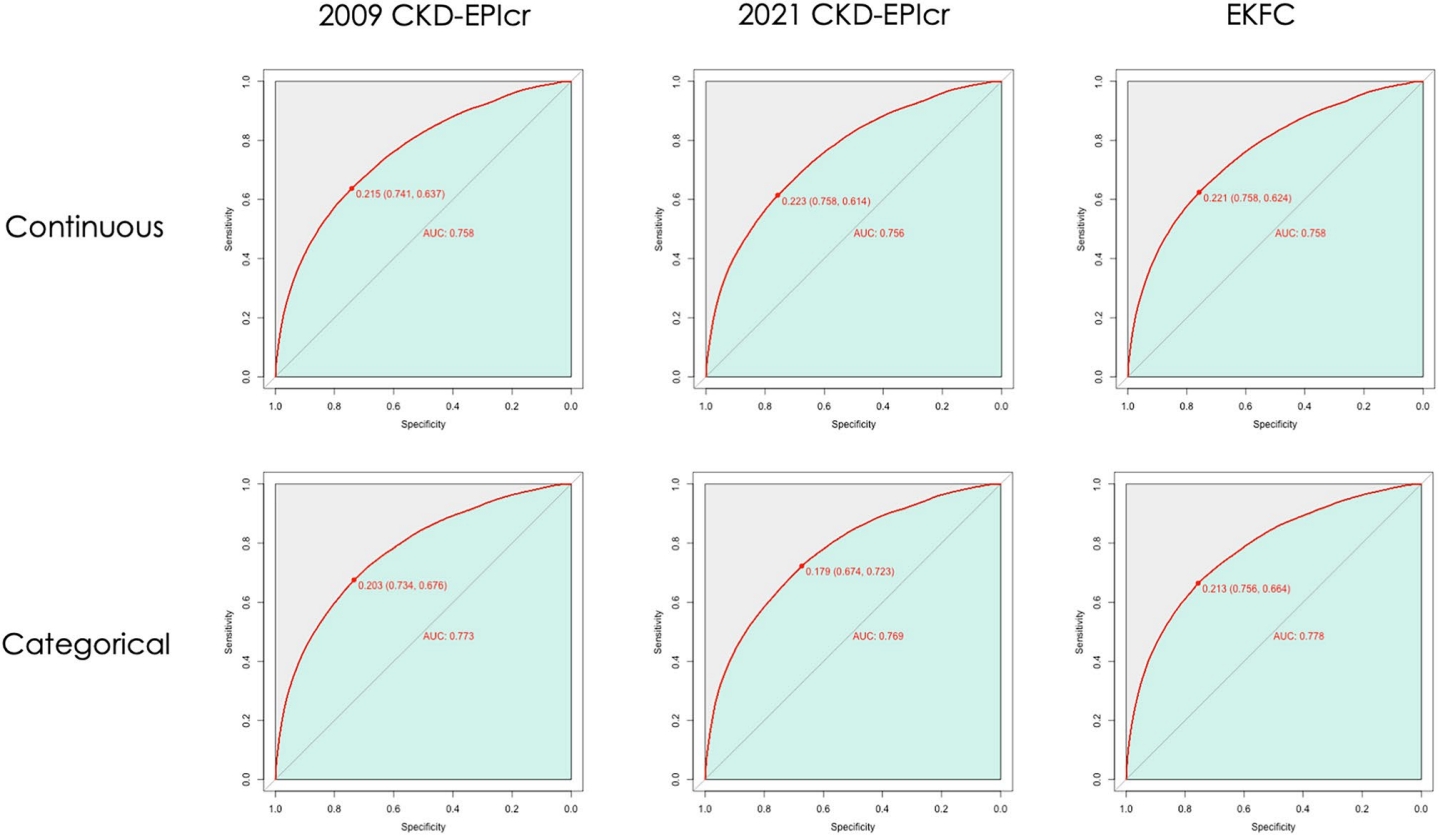
Receiver operating curves (ROC) for prediction of AKI (multivariable-model 2). In continuous and categorical eGFR, the performance of the 2009 and 2021 CKD-EPIcr and EKFC equation models for predicting AKI within 7 days from baseline was evaluated by AUC. For AKI, the 2009 CKD-EPIcr model had significantly better predictive power than the 2021 CKD-EPIcr model for both continuous (model 2, 0.7583 [0.755–0.7617] vs. 0.7564 [0.7531–0.7597]; *P* < 0.001) and categorical eGFR (model 2, 0.7735 [0.7702–0.7767] vs. 0.7694 [0.7662–0.7727]; < 0.001). The blue shaded area represents the AUC, and the circle on each curve represents the optimal cutoff value. AKI, acute kidney injury; eGFR, estimated glomerular filtration rate; AUC, area under the curve

As the main analysis, we evaluated the performance of AKI risk stratification models according to the three GFR-estimating equations using AUROC (Table 3, Fig. 3). Because kidney function is commonly evaluated using eGFR categories in various clinical settings, we assessed the performance of the models using both continuous and categorical eGFR values.

**Table 3:** Performance of GFR estimating equations in classifying AKI using ROC-AUC

Continuous eGFR				
	AUC	^c^Comparison of AUC		
	Model 1^a^	A vs. B	A vs. C	B vs. C
2009 CKD-EPIcr	0.6328 (0.6288–0.6368)	< 0.001	< 0.001	< 0.001
2021 CKD-EPIcr	0.6195 (0.6156–0.6234)			
EKFC	0.63 (0.6261–0.634)			
	Model 2^b^	A vs. B	A vs. C	B vs. C
2009 CKD-EPIcr	0.7583 (0.755–0.7617)	< 0.001	< 0.001	< 0.001
2021 CKD-EPIcr	0.7564 (0.7531–0.7597)			
EKFC	0.7577 (0.7543 – 0.761)			

Categorical eGFR				
	AUC	Comparison of AUC		
	Model 1	A vs. B	A vs. C	B vs. C
2009 CKD-EPIcr	0.6685 (0.6647–0.6723)	< 0.001	< 0.001	< 0.001
2021 CKD-EPIcr	0.6533 (0.6495–0.6571)			
EKFC	0.68 (0.6762–0.6838)			
	Model 2	A vs. B	A vs. C	B vs. C
2009 CKD-EPIcr	0.7735 (0.7702–0.7767)	< 0.001	< 0.001	< 0.001
2021 CKD-EPIcr	0.7694 (0.7662–0.7727)			
EKFC	0.7779 (0.7747–0.7811)			

*AKI* acute kidney injury, *eGFR* estimated glomerular filtration rate, *ROC* receiver operating characteristic, *AUC* area under the curve

^a^Multivariable model 1: adjusted with sex, age, and body mass index

^b^Multivariable model 2: adjusted for sex, age, hospital visit type, department, body mass index, systolic blood pressure, hemoglobin, serum sodium, procedure within 2 weeks before the baseline date, history of hypertension, diabetes mellitus, and malignancy

^c^DeLong test for two correlated ROC curves; A, B, and C refer to the following equations: A = 2009 CKD-EPIcr, B = 2021 CKD-EPIcr, and C = EKFC

In the discrimination of AKI, the 2009 CKD-EPIcr-fitted model showed better performance than either the 2021 CKD-EPIcr or EKFC models for continuous eGFR. In the multivariable-adjusted model, the AUROC of the 2009 CKD-EPIcr model (model 2, 0.7583 [0.755–0.7617]) was higher than that of the 2021 CKD-EPIcr model (model 2, 0.7564 [0.7531–0.7597]) and the EKFC model (model 2, 0.7577 [0.7543–0.761]). A pairwise comparison of AUROC values showed significant differences (*P* < 0.001) in the performance of the three models. The results of the categorical eGFR-fitted prediction models were consistent with those of the continuous models. The AUROC was significantly lower in the 2021 CKD-EPIcr model (model 2, 0.7694 [0.7662–0.7727]) than in the 2009 CKD-EPIcr (model 2, 0.7735 [0.7702–0.7767]) and EKFC (model 2, 0.7779 [0.7747–0.7811]) models. (2021 CKD-EPIcr vs. 2009 CKD-EPIcr or EKFC, *P* < 0.001).

### Reclassification of AKI

**Fig. 4:**
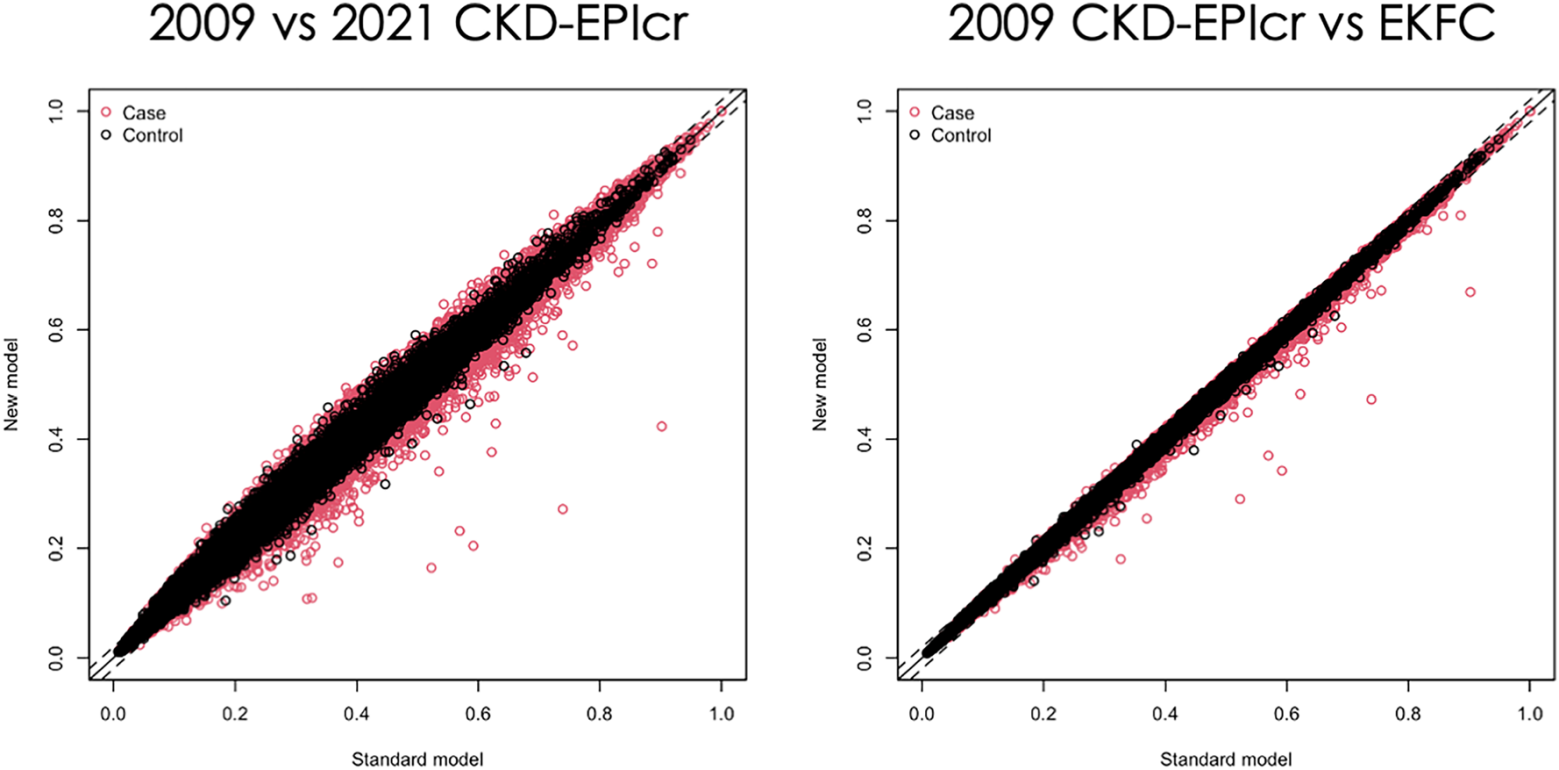
Net reclassification improvement for AKI (multivariable-model 2). Reclassification plots. The 2009 CKD-EPIcr equation model was set as the standard model for all plots, and either the 2021 CKD-EPIcr or EKFC model was set as the new model

To assess the improvement in classifying participants into correct groups according to AKI occurrence, multivariable-adjusted net reclassification improvement was performed for both the 2021 CKD-EPIcr and EKFC models against the 2009 CKD-EPIcr model (Table 4, Fig. 4). In the reclassification of AKI, both the 2021 CKD-EPIcr (− 7.24 [− 8.21–− 6.21]) and EKFC models (− 2.38 [− 2.72–− 1.97]) showed worse classification performances than the 2009 CKD-EPIcr model. Specifically, the proportion of participants correctly reclassified as having AKI decreased by 4.43 (3.46–5.54) % and 2.40 (1.99–2.75) % in the 2021 CKD-EPIcr and EKFC models, respectively, compared to the 2009 CKD-EPIcr model. In other words, the 2021 CKD-EPIcr model misclassified a median of 1,136 patients with AKI into the non-AKI group, and the EKFC model misclassified a median of 615 patients with AKI into the non-AKI group.

**Table 4:** Reclassification of AKI events and non-events comparing between 2009 CKD-EPIcr equation and either 2021 CKD-EPIcr or EKFG equation

	Proportion of participants correctly reclassified		NRI (95% CI)	AKI reclassified (N)
	AKI reclassified, % (95% CI)	Non-AKI reclassified, % (95% CI)		
2009 CKD-EPIcr vs				
2021 CKD-EPIcr	− 4.43 (− 5.54–− 3.46)	− 2.8 (− 2.94–− 2.64)	− 7.24 (− 8.21–− 6.21)	1136 (887–1421)
EKFC	− 2.40 (− 2.75–− 1.99)	0.02 (0.00–0.04)	− 2.38 (− 2.72–− 1.97)	615 (510–705)

In all models, continuous eGFR variables were used

All models were multivariate-adjusted for sex, age, hospital visit type, department, body mass index, systolic blood pressure, hemoglobin, serum sodium, procedure within 2 weeks before the baseline date, history of hypertension, diabetes mellitus, and malignancy

*AKI* acute kidney injury, *N RI* net reclassification index

### Subgroup analyses

The discriminative performance for AKI evaluated using AUROC in various subgroups is presented in Supplemental Tables 2 and 3 for continuous and categorical eGFR, respectively. In the sex subgroups, the multivariable model fitted with the 2021 CKD-EPIcr equation had significantly worse performance in the discrimination of AKI than the models fitted with the 2009 CKD-EPIcr or EKFC equations, regardless of whether eGFR was continuous or categorical. The DM, age, and procedural subgroups revealed similar results to those of the sex subgroups, exhibiting worse AKI discriminative performance with the use of the 2021 CKD-EPIcr equation compared to the other two equations for continuous and categorical eGFR. In patients with a higher baseline eGFR, the 2021 CKD-EPIcr model showed worse performance in discriminating AKI, whereas in patients with a lower baseline eGFR, there was no significant difference among the three eGFR equation-fitted models. The poor discriminative power of the 2021 CKD-EPIcr model was still observed when stratified into subgroups based on the years when the SCr examination was conducted.

In addition to assessing the discriminative performance of the three equations for AKI using the AUROC in the subgroups, we also evaluated their reclassification power using continuous eGFR (Supplemental Table 4). In most subgroups, the reclassification power for AKI was significantly decreased in the 2021 CKD-EPIcr and EKFC models compared with the 2009 CKD-EPIcr model. Negative net reclassification improvement results were observed in the sex, DM, younger age, higher baseline eGFR, and procedural procedure and year subgroups. The lower baseline eGFR subgroups did not show significant differences in reclassification between the three-equation models.

## Discussion

In this study, we compared the discriminative performance of three eGFR equations for AKI risk on a large scale; the sample comprised mostly East Asians. We identified significant differences in the performance of the AKI risk stratification models according to the GFR estimation equation. In the 2009 CKD-EPIcr equation-fitted model, significantly better performance in discriminating AKI was exhibited compared with the models developed using the 2021 CKD-EPIcr or EKFC equations. Thus, our findings suggest that the 2009 CKD-EPIcr equation may be a preferable option for estimating GFR in patients who visit medical services compared to the other two equations.

Several studies have evaluated the clinical implications associated with the transition from the 2009 to 2021 CKD-EPIcr equations. There was a 3 to 5 mL/min/1.73 m^2^ increase in eGFR, and up to 30% of individuals with lower eGFR were reclassified into the higher KDIGO GFR category after transitioning from the 2009 to the 2021 CKD-EPIcr equation in non-blacks [12, 25–28]. Moreover, patients who were reclassified to a higher eGFR category using the 2021 CKD-EPI equation demonstrated a higher risk of mortality and cardiovascular outcomes compared to those who were not reclassified [12]. Nevertheless, the association between eGFR and kidney, cardiovascular, and mortality outcomes did not show significant differences between the two equations [12]. However, due to limited data on the effects of the transition of eGFR equations, further research is necessary to determine the impact of the changes in eGFR on clinical practice and outcomes, and additional evidence is especially required for Asian populations.

The eGFR is used not only for mid- to long-term outcome prediction, but also for short-term risk assessment, such as AKI, and research is needed to identify the best eGFR equation for AKI risk stratification to provide new insights into the impact of eGFR equations in AKI risk assessment.

Our study has several strengths. First, we demonstrated the effect of transitioning eGFR equations on the prediction and reclassification of AKI using multivariate regression models that included baseline eGFR values. Given that an increase in baseline GFR estimates does not indicate an improvement in the underlying kidney function, understanding the impact of the alteration of GFR estimates on kidney outcomes is important. Second, as we used two recently developed GFR-estimating equations against the current standard equation to compare the prediction and reclassification power for AKI, our findings may serve as a reference for deciding whether to adopt new eGFR equations as well as guidance for baseline eGFR-based medical practices. Third, our study included a large-scale cohort of 187,139 patients, mostly East Asian, among whom 27,447 experienced at least one AKI event. The study was not limited to hospitalized patients, but also included patients who visited outpatient clinics or emergency rooms, thereby expanding the scope of our findings and conferring statistical power for the reclassification and prediction of AKI. In addition, as previous studies mostly lacked Asian individuals, this study provides evidence from East Asian nations.

While AKI is diagnosed with the increase of serum creatinine level, the AKI risk stratification is frequently assessed with eGFR value as several studies suggested its value as a predictive marker [29–32]. However, the difference between the eGFR value calculated by the 2009 and 2021 CKD-EPIcr equations challenges clinicians to decide which eGFR equation to use for more precise evaluation of the risk of AKI in patients [12, 33]. Depending on the eGFR equation, the pattern of early prediction and intervention of AKI may substantially change. Furthermore, because the eGFR threshold is used as a criterion for determining various medical practices, the AKI-associated long-term outcomes may differ depending on the eGFR equations. Serum and urinary biomarkers, including neutrophil gelatinase-associated lipocalin or kidney injury molecule 1, are indicators of AKI with good predictive value, however they are associated with specific pathological processes that mediate AKI, resulting in limited generalizability and utility in clinical practice [17]. Also, current studies of AKI prediction or risk stratification are mostly focused on specific clinical settings, including perioperative, critical illness, or radiocontrast [34], and the risk prediction models or stratification scores are rarely used in real-time to predict which patients are at high risk [34]. Estimated GFR is an established predictor of AKI that is easily calculated and universally used in the clinical field [35], and therefore, it is crucial to fully consider the clinical implications of changes of eGFR equation and evaluate the AKI prediction and reclassification power of different eGFR equations.

In our study, we found that the AKI discriminating power of the 2021 CKD-EPIcr and EKFC equations for AKI was significantly lower than that of the 2009 CKD-EPIcr equation, as shown in the AUROC between the prediction models. Furthermore, net reclassification improvement analysis showed negative reclassification performance of AKI in both the 2021 CKD-EPIcr and EKFC models, indicating that there were a significant number of misclassifications of AKI as non-AKI in comparison to the 2009 CKD-EPIcr model. Our study showed that certain subgroups at high risk of kidney outcomes, including male sex, DM, and procedural subgroups, may benefit from maintaining the 2009 CKD-EPIcr equations [17, 36, 37]. Notably, participants with higher eGFR showed significantly worse discrimination for AKI in the prediction model using the 2021 CKD-EPIcr equation. Because SCr does not linearly correlate with GFR, patients with higher GFR need a large degree of change in GFR to exhibit a measurable change in SCr [38]. Even subtle increases in SCr levels may indicate a substantial loss of functioning nephrons [39], resulting in significant long-term sequelae or adverse outcomes, particularly in those with higher GFR [40–43]. Moreover, patients with eGFR ≥ 60 and < 90 mL/min/1.73 m^2^ are at a critical point for clinical decisions such as the diagnosis of CKD, and since there is currently no effective therapy to reverse the loss of kidney function, early prediction and intervention for AKI prior to its onset are important in this subgroup. The lower predictive power for AKI by the 2021 CKD-EPIcr equation could be particularly problematic in these patients. Thus, our study suggests that the adoption of the 2021 CKD-EPIcr equation may affect the misprediction of AKI owing to changes in baseline eGFR values, which can result in delayed access to preventative or therapeutic interventions for AKI despite its sociocultural benefits.

Recent publications reported that the 2021 CKD-EPI equation did not show superior predictability for major clinical outcomes including death, cardiovascular event, and kidney failure compared to the 2009 CKD-EPI equation in the Asian population [44, 45], and lower accuracy of the 2021 CKD-EPI equation was identified in both Korean and Chinese cohorts [33, 46]. Moreover, several Asian countries including Korea, China, India, Singapore and Russia are expected to have underestimated the prevalence of CKD, which is unlikely to improve clinical outcomes in the Asian population [47]. Considering the findings of our study and the current evidence, we suggest maintaining the use of the 2009 CKD-EPI equation in Asian populations and propose gathering further data regarding the association of the 2021 CKD-EPI equation with various outcomes, for further discussion.

Our study has some limitations. First, a selection bias may have existed because the study was conducted at a single center, and the requirement was to have consecutive SCr measurements. Second, information on urine output and long-term cardiovascular prognosis was lacking. Third, the issue of generalizability should be noted because this was a single-ethnic study. The differences may be even larger in patients of black ethnicity considering the formula of the 2009 CKD-EPIcr equation.

In summary, we found that AKI risk stratification based on the 2009 CKD-EPIcr equation showed a better discriminative performance than the 2021 CKD-EPIcr or EKFC equations in a cohort that included mostly Asian individuals. Acceptance of the 2021 CKD-EPIcr equation should be considered after a thorough evaluation of its potential clinical impact resulting from a change in the GFR estimating equation. Further studies in various populations are warranted to establish the external validity of these findings.

### Supplementary Information

Below is the link to the electronic supplementary material.

Supplementary file1 (PDF 258 kb)

## Data Availability

The datasets were analyzed in the current study and are available from the corresponding author upon reasonable request.
