# Supplementary material for: Evaluation of risk stratification for acute kidney injury: a comparative analysis of EKFC, 2009 and 2021 CKD-EPI glomerular filtration estimating equations
Source: J Nephrol. 2024 Feb 12;37(3):681–93. doi: 10.1007/s40620-023-01883-7 (PMC11150313; doi:10.1007/s40620-023-01883-7)
Supplement: Supplementary file 1 — Supplementary file1 (PDF 258 kb) [file 40620_2023_1883_MOESM1_ESM.pdf]

Supplemental data 1. Definition of comorbidities and medications

Comorbidities	ICD-10 code
Hypertension	I10-12, I15, H350
Diabetes mellitus	E10-11, E13-14, T861
Malignancy	C00-14, C15-26, C30-39, C40-41, C43-44, C45-49, C50, C51-58, C60-63, C64-68, C69-72, C73-75, C76-80, C7A, C7B, C81-96
**Drug**	**ATC code**
Angiotensin receptor blocker	C09CA, C09D, C10BX10, C10BX16
Angiotensin-converting enzyme inhibitor	C09AA, C09B, C10BX04, C10BX06, C10BX07, C10BX12~15, 17, 18
Beta-blockers	C07
Calcium channel blocker	C08, C09, C04FB, C05CX
Diuretics	C03, C09DA
Metformin	A10BA02
Insulin	A10AB, A10AC, A10AD, A10AE, A10AF
Blood glucose lowering agents	A10B

ICD = International Classification of Disease, ATC = Anatomical Therapeutic Chemical

Supplemental table 2. Subgroup analysis of AKI-classifying performance of eGFR equations using ROC-AUC (continuous eGFR)

	AUC	*Comparison of AUC		
**Male**				
	Model 1[a]	A vs. B	A vs. C	B vs. C
2009 CKD-EPIcr	0.6201 (0.6148 – 0.6254)	<0.001	<0.001	<0.001
2021 CKD-EPIcr	0.6112 (0.6061 – 0.6164)			
EKFC	0.6141 (0.6089 – 0.6193)			
	Model 2[b]	A vs. B	A vs. C	B vs. C
2009 CKD-EPIcr	0.7466 (0.742 – 0.7512)	<0.001	<0.001	0.012
2021 CKD-EPIcr	0.7455 (0.7409 – 0.7501)			
EKFC	0.7457 (0.7412 – 0.7503)			
**Female**				
	Model 1	A vs. B	A vs. C	B vs. C
2009 CKD-EPIcr	0.648 (0.6417 – 0.6543)	<0.001	<0.001	<0.001
2021 CKD-EPIcr	0.6237 (0.6176 – 0.6298)			
EKFC	0.6515 (0.6452 – 0.6578)			
	Model 2	A vs. B	A vs. C	B vs. C
2009 CKD-EPIcr	0.7755 (0.7705 – 0.7804)	<0.001	<0.001	<0.001
2021 CKD-EPIcr	0.7728 (0.7679 – 0.7776)			
EKFC	0.7757 (0.7707 – 0.7806)			
**DM**				
	Model 1	A vs. B	A vs. C	B vs. C
2009 CKD-EPIcr	0.5972 (0.587 – 0.6074)	0.018	0.05	0.008
2021 CKD-EPIcr	0.6006 (0.5903 – 0.6109)			
EKFC	0.5985 (0.5883 – 0.6088)			
	Model 2	A vs. B	A vs. C	B vs. C
2009 CKD-EPIcr	0.7677 (0.7593 – 0.7761)	<0.001	<0.001	<0.001
2021 CKD-EPIcr	0.7698 (0.7614 – 0.7781)			
EKFC	0.7685 (0.7601 – 0.7769)			
**Non-DM**				
	Model 1	A vs. B	A vs. C	B vs. C
2009 CKD-EPIcr	0.6435 (0.639 – 0.648)	<0.001	<0.001	<0.001
2021 CKD-EPIcr	0.6248 (0.6204 – 0.6291)			
EKFC	0.6413 (0.6368 – 0.6457)			
	Model 2	A vs. B	A vs. C	B vs. C
2009 CKD-EPIcr	0.7524 (0.7487 – 0.7562)	<0.001	<0.001	<0.001
2021 CKD-EPIcr	0.7495 (0.7458 – 0.7531)			
EKFC	0.7517 (0.748 – 0.7554)			
**Age ≥ 65**				
	Model 1	A vs. B	A vs. C	B vs. C
2009 CKD-EPIcr	0.5698 (0.564 – 0.5756)	0.962	0.6238	0.4502
2021 CKD-EPIcr	0.5698 (0.5639 – 0.5757)			
EKFC	0.5701 (0.5642 – 0.576)			
	Model 2	A vs. B	A vs. C	B vs. C
2009 CKD-EPIcr	0.7421 (0.7373 – 0.7469)	<0.001	0.002	<0.001
2021 CKD-EPIcr	0.743 (0.7382 – 0.7478)			
EKFC	0.7425 (0.7377 – 0.7473)			
**Age < 65**				
	Model 1	A vs. B	A vs. C	B vs. C
2009 CKD-EPIcr	0.6043 (0.5983 – 0.6102)	<0.001	<0.001	<0.001
2021 CKD-EPIcr	0.5828 (0.5771 – 0.5884)			
EKFC	0.5987 (0.5929 – 0.6046)			
	Model 2	A vs. B	A vs. C	B vs. C
2009 CKD-EPIcr	0.7571 (0.7522 – 0.7619)	<0.001	<0.001	0.093

2021 CKD-EPIcr	0.755 (0.7503 – 0.7598)			
EKFC	0.7564 (0.7515 – 0.7612)			
**eGFR < 60 mL/min/1.73m²**				
	Model 1	A vs. B	A vs. C	B vs. C
2009 CKD-EPIcr	0.6807 (0.6716 – 0.6898)	0.049	0.599	0.379
2021 CKD-EPIcr	0.6808 (0.6716 – 0.6899)			
EKFC	0.6806 (0.6715 – 0.6898)			
	Model 2	A vs. B	A vs. C	B vs. C
2009 CKD-EPIcr	0.7563 (0.7481 – 0.7645)	0.027	0.655	0.3938
2021 CKD-EPIcr	0.7563 (0.7481 – 0.7646)			
EKFC	0.7562 (0.748 – 0.7645)			
**eGFR ≥ 60 mL/min/1.73m²**				
	Model 1	A vs. B	A vs. C	B vs. C
2009 CKD-EPIcr	0.7274 (0.7229 – 0.7319)	<0.001	<0.001	<0.001
2021 CKD-EPIcr	0.7216 (0.7171 – 0.7261)			
EKFC	0.7286 (0.7242 – 0.7331)			
	Model 2	A vs. B	A vs. C	B vs. C
2009 CKD-EPIcr	0.7908 (0.7871 – 0.7946)	<0.001	<0.001	<0.001
2021 CKD-EPIcr	0.7856 (0.7819 – 0.7894)			
EKFC	0.7903 (0.7865 – 0.794)			
**Procedure**				
	Model 1	A vs. B	A vs. C	B vs. C
2009 CKD-EPIcr	0.6668 (0.6596 – 0.6739)	<0.001	<0.001	<0.001
2021 CKD-EPIcr	0.6472 (0.6402 – 0.6543)			
EKFC	0.6652 (0.6581 – 0.6723)			
	Model 2	A vs. B	A vs. C	B vs. C
2009 CKD-EPIcr	0.7028 (0.6961 – 0.7095)	<0.001	<0.001	<0.001
2021 CKD-EPIcr	0.691 (0.6843 – 0.6976)			
EKFC	0.7016 (0.6949 – 0.7083)			
**No procedure**				
	Model 1	A vs. B	A vs. C	B vs. C
2009 CKD-EPIcr	0.6268 (0.6221 – 0.6315)	<0.001	<0.001	<0.001
2021 CKD-EPIcr	0.6214 (0.6167 – 0.626)			
EKFC	0.6247 (0.6201 – 0.6294)			
	Model 2	A vs. B	A vs. C	B vs. C
2009 CKD-EPIcr	0.7879 (0.7839 – 0.7918)	0.076	0.4283	0.022
2021 CKD-EPIcr	0.7883 (0.7844 – 0.7923)			
EKFC	0.788 (0.784 – 0.7919)			
**2011–2013**				
	Model 1	A vs. B	A vs. C	B vs. C
2009 CKD-EPIcr	0.6026 (0.5933 – 0.6118)	<0.001	<0.001	<0.001
2021 CKD-EPIcr	0.5897 (0.5809 – 0.5985)			
EKFC	0.5986 (0.5895 – 0.6077)			
	Model 2	A vs. B	A vs. C	B vs. C
2009 CKD-EPIcr	0.7534 (0.7455 – 0.7614)	0.004	<0.001	0.013
2021 CKD-EPIcr	0.7519 (0.7439 – 0.7599)			
EKFC	0.7529 (0.7449 – 0.7608)			
**2014–2017**				
	Model 1	A vs. B	A vs. C	B vs. C
2009 CKD-EPIcr	0.6545 (0.6484 – 0.6607)	<0.001	<0.001	<0.001
2021 CKD-EPIcr	0.637 (0.631 – 0.6431)			
EKFC	0.6513 (0.6452 – 0.6574)			
	Model 2	A vs. B	A vs. C	B vs. C
2009 CKD-EPIcr	0.7828 (0.7778 – 0.7877)	<0.001	<0.001	<0.001
2021 CKD-EPIcr	0.7804 (0.7755 – 0.7853)			
EKFC	0.7819 (0.777 – 0.7869)			
**2018–2021**				
	Model 1	A vs. B	A vs. C	B vs. C

2009 CKD-EPIcr	0.6389 (0.6326 – 0.6453)	<0.001	<0.001	<0.001
2021 CKD-EPIcr	0.6325 (0.6262 – 0.6388)			
EKFC	0.6374 (0.6311 – 0.6438)			
	Model 2	A vs. B	A vs. C	B vs. C
2009 CKD-EPIcr	0.7685 (0.7632 – 0.7739)	0.449	0.051	0.773
2021 CKD-EPIcr	0.7683 (0.763 – 0.7736)			
EKFC	0.7684 (0.763 – 0.7737)			

AKI = acute kidney injury, eGFR = estimated glomerular filtration rate, ROC = receiver operating characteristic, AUC = area under curve, DM = diabetes mellitus

[a]Multivariable model 1: adjusted with sex, age, and body mass index

[b]Multivariable model 2: adjusted with sex, age, hospital visit type, department, body mass index, systolic blood pressure, hemoglobin, serum sodium, procedure within 2 weeks before baseline date, history of hypertension, diabetes mellitus, and malignancy

* DeLong's test for two correlated ROC curves; A, B, and C refers to following equations: A = 2009 CKD-EPIcr, B = 2021 CKD-EPIcr, C = EKFC

Supplemental table 3. Subgroup analysis of AKI-classifying performance of eGFR equations using ROC-AUC (categorical eGFR)

	AUC	*Comparison of AUC		
**Male**				
	Model 1[a]	A vs. B	A vs. C	B vs. C
2009 CKD-EPIcr	0.6605 (0.6554 – 0.6656)	<0.001	<0.001	<0.001
2021 CKD-EPIcr	0.6463 (0.6412 – 0.6514)			
EKFC	0.6684 (0.6633 – 0.6735)			
	Model 2[b]	A vs. B	A vs. C	B vs. C
2009 CKD-EPIcr	0.7613 (0.7568 – 0.7658)	<0.001	<0.001	<0.001
2021 CKD-EPIcr	0.7577 (0.7531 – 0.7622)			
EKFC	0.7648 (0.7603 – 0.7693)			
**Female**				
	Model 1	A vs. B	A vs. C	B vs. C
2009 CKD-EPIcr	0.6749 (0.6691 – 0.6806)	<0.001	<0.001	<0.001
2021 CKD-EPIcr	0.6566 (0.6507 – 0.6625)			
EKFC	0.6914 (0.6856 – 0.6972)			
	Model 2	A vs. B	A vs. C	B vs. C
2009 CKD-EPIcr	0.7902 (0.7855 – 0.7949)	<0.001	<0.001	<0.001
2021 CKD-EPIcr	0.7861 (0.7814 – 0.7908)			
EKFC	0.7954 (0.7907 – 0.8001)			
**DM**				
	Model 1	A vs. B	A vs. C	B vs. C
2009 CKD-EPIcr	0.6828 (0.6732 – 0.6925)	<0.001	0.463	<0.001
2021 CKD-EPIcr	0.667 (0.6572 – 0.6767)			
EKFC	0.6854 (0.6756 – 0.6951)			
	Model 2	A vs. B	A vs. C	B vs. C
2009 CKD-EPIcr	0.7946 (0.7866 – 0.8026)	0.003	0.136	<0.001
2021 CKD-EPIcr	0.7918 (0.7838 – 0.7998)			
EKFC	0.7964 (0.7884 – 0.8044)			
**Non-DM**				
	Model 1	A vs. B	A vs. C	B vs. C
2009 CKD-EPIcr	0.6598 (0.6556 – 0.6639)	<0.001	<0.001	<0.001
2021 CKD-EPIcr	0.6437 (0.6395 – 0.6479)			
EKFC	0.6731 (0.669 – 0.6773)			
	Model 2	A vs. B	A vs. C	B vs. C
2009 CKD-EPIcr	0.7653 (0.7618 – 0.7689)	<0.001	<0.001	<0.001
2021 CKD-EPIcr	0.7608 (0.7572 – 0.7644)			
EKFC	0.7704 (0.7669 – 0.774)			
**Age ≥ 65**				
	Model 1	A vs. B	A vs. C	B vs. C
2009 CKD-EPIcr	0.6808 (0.6756 – 0.6861)	<0.001	<0.001	<0.001
2021 CKD-EPIcr	0.6477 (0.6422 – 0.6531)			
EKFC	0.6984 (0.693 – 0.7039)			
	Model 2	A vs. B	A vs. C	B vs. C
2009 CKD-EPIcr	0.7729 (0.7683 – 0.7775)	<0.001	<0.001	<0.001
2021 CKD-EPIcr	0.7635 (0.7588 – 0.7682)			
EKFC	0.7873 (0.7827 – 0.7918)			
**Age < 65**				
	Model 1	A vs. B	A vs. C	B vs. C
2009 CKD-EPIcr	0.686 (0.676 – 0.696)	0.036	<0.001	<0.001
2021 CKD-EPIcr	0.685 (0.675 – 0.695)			
EKFC	0.689 (0.679 – 0.699)			
	Model 2	A vs. B	A vs. C	B vs. C
2009 CKD-EPIcr	0.791 (0.782 – 0.799)	0.256	0.037	0.314

2021 CKD-EPIcr	0.791 (0.783 – 0.800)			
EKFC	0.791 (0.783 – 0.800)			
**eGFR < 60 mL/min/1.73m²**				
	Model 1	A vs. B	A vs. C	B vs. C
2009 CKD-EPIcr	0.6746 (0.6654 – 0.6838)	0.6078	0.3414	0.2224
2021 CKD-EPIcr	0.6737 (0.6645 – 0.6829)			
EKFC	0.6763 (0.6671 – 0.6855)			
	Model 2	A vs. B	A vs. C	B vs. C
2009 CKD-EPIcr	0.753 (0.7447 – 0.7612)	0.9808	0.2145	0.3361
2021 CKD-EPIcr	0.753 (0.7447 – 0.7613)			
EKFC	0.7541 (0.7459 – 0.7624)			
**eGFR ≥ 60 mL/min/1.73m²**				
	Model 1	A vs. B	A vs. C	B vs. C
2009 CKD-EPIcr	0.6477 (0.6434 – 0.652)	<0.001	<0.001	<0.001
2021 CKD-EPIcr	0.6273 (0.623 – 0.6316)			
EKFC	0.6703 (0.666 – 0.6746)			
	Model 2	A vs. B	A vs. C	B vs. C
2009 CKD-EPIcr	0.7679 (0.7642 – 0.7715)	<0.001	<0.001	<0.001
2021 CKD-EPIcr	0.7626 (0.759 – 0.7663)			
EKFC	0.7754 (0.7718 – 0.7791)			
**Procedure**				
	Model 1	A vs. B	A vs. C	B vs. C
2009 CKD-EPIcr	0.6562 (0.6492 – 0.6631)	<0.001	<0.001	<0.001
2021 CKD-EPIcr	0.6413 (0.6343 – 0.6483)			
EKFC	0.6779 (0.671 – 0.6848)			
	Model 2	A vs. B	A vs. C	B vs. C
2009 CKD-EPIcr	0.6973 (0.6907 – 0.7039)	<0.001	<0.001	<0.001
2021 CKD-EPIcr	0.6885 (0.6817 – 0.6952)			
EKFC	0.7125 (0.7059 – 0.719)			
**No procedure**				
	Model 1	A vs. B	A vs. C	B vs. C
2009 CKD-EPIcr	0.6802 (0.6757 – 0.6848)	<0.001	<0.001	<0.001
2021 CKD-EPIcr	0.6662 (0.6616 – 0.6707)			
EKFC	0.6889 (0.6843 – 0.6934)			
	Model 2	A vs. B	A vs. C	B vs. C
2009 CKD-EPIcr	0.8033 (0.7995 – 0.8072)	<0.001	<0.001	<0.001
2021 CKD-EPIcr	0.8009 (0.797 – 0.8047)			
EKFC	0.8058 (0.8019 – 0.8096)			
**2011–2013**				
	Model 1	A vs. B	A vs. C	B vs. C
2009 CKD-EPIcr	0.6556 (0.6469 – 0.6642)	<0.001	<0.001	<0.001
2021 CKD-EPIcr	0.6385 (0.6297 – 0.6473)			
EKFC	0.6675 (0.6589 – 0.6762)			
	Model 2	A vs. B	A vs. C	B vs. C
2009 CKD-EPIcr	0.7712 (0.7635 – 0.779)	<0.001	<0.001	<0.001
2021 CKD-EPIcr	0.7665 (0.7586 – 0.7744)			
EKFC	0.777 (0.7694 – 0.7846)			
**2014–2017**				
	Model 1	A vs. B	A vs. C	B vs. C
2009 CKD-EPIcr	0.6769 (0.6711 – 0.6826)	<0.001	<0.001	<0.001
2021 CKD-EPIcr	0.6605 (0.6547 – 0.6664)			
EKFC	0.6869 (0.6812 – 0.6927)			
	Model 2	A vs. B	A vs. C	B vs. C
2009 CKD-EPIcr	0.7961 (0.7913 – 0.8008)	<0.001	<0.001	<0.001
2021 CKD-EPIcr	0.7919 (0.7871 – 0.7967)			
EKFC	0.7991 (0.7944 – 0.8039)			
**2018–2021**				
	Model 1	A vs. B	A vs. C	B vs. C

2009 CKD-EPIcr	0.6784 (0.6722 – 0.6847)	<0.001	<0.001	<0.001
2021 CKD-EPIcr	0.6653 (0.659 – 0.6716)			
EKFC	0.6913 (0.685 – 0.6975)			
	Model 2	A vs. B	A vs. C	B vs. C
2009 CKD-EPIcr	0.7834 (0.7782 – 0.7886)	<0.001	<0.001	<0.001
2021 CKD-EPIcr	0.7802 (0.775 – 0.7855)			
EKFC	0.7879 (0.7826 – 0.7931)			

AKI = acute kidney injury, eGFR = estimated glomerular filtration rate, ROC = receiver operating characteristic, AUC = area under curve, DM = diabetes mellitus

[a]Multivariable model 1: adjusted with sex, age, and body mass index

[b]Multivariable model 2: adjusted with sex, age, hospital visit type, department, body mass index, systolic blood pressure, hemoglobin, serum sodium, procedure within 2 weeks before baseline date, history of hypertension, diabetes mellitus, and malignancy

* DeLong's test for two correlated ROC curves; A, B, and C refers to following equations: A = 2009 CKD-EPIcr, B = 2021 CKD-EPIcr, C = EKFC

Supplemental table 4. Subgroup analysis of net reclassification improvement of AKI between eGFR equations

	Proportion of participants correctly reclassified		NRI (95% CI)
	AKI reclassified, % (95% CI)	Non-AKI reclassified, % (95% CI)	
**Male**			
2009 CKD-EPIcr vs.			
2021 CKD-EPIcr	-1.15 (-2.17 – 0.12)	-2.64 (-2.89 – -2.38)	-3.79 (-4.72 – -2.51)
EKFC	-0.16 (-1.72 – 1.09)	-1.18 (-1.37 – -0.90)	-1.34 (-2.79 – -0.25)
**Female**			
2009 CKD-EPIcr vs.			
2021 CKD-EPIcr	-11.5 (-14.5 – -10.6)	-2.9 (-3.02 – -2.81)	-14.5 (-17.5 – -13.5)
EKFC	0.02 (-0.04 – 0.03)	0.00 (0.00 – 0.00)	-0.02 (-0.04 – 0.03)
**DM**			
2009 CKD-EPIcr vs.			
2021 CKD-EPIcr	2.21 (0.11 – 4.36)	-5.51 (-5.84 - -5.10)	-3.3 (-5.45 – -1.38)
EKFC	-0.57 (-1.54 – 0.56)	-0.07 (-0.22 - 0.00)	-0.65 (-1.63 – -0.42)
**No DM**			
2009 CKD-EPIcr vs.			
2021 CKD-EPIcr	-8.34 (-8.4 – -6.1)	2.7 (-2.8 – -2.6)	-11.05 (-11.13 – -8.9)
EKFC	-3.65 (-3.79 – -3.31)	0.00 (0.00 – 0.00)	-3.59 (-3.72 – -3.25)
**Age ≥ 65**			
2009 CKD-EPIcr vs.			
2021 CKD-EPIcr	7.69 (7.29 – 9.13)	-3.13 (-3.26 – -3.10)	4.56 (4.17 – 5.87)
EKFC	1.82 (1.38 – 2.17)	-0.29 (-0.32 – -0.18)	1.53 (1.19 – 1.85)
**Age < 65**			
2009 CKD-EPIcr vs.			
2021 CKD-EPIcr	-9.44 (-10.54 – -8.92)	-1.02 (-1.12 – -0.86)	-10.47 (-11.48 – -9.84)
EKFC	-5.24 (-5.85 – -4.45)	0.58 (0.47 – 0.78)	-4.65 (-5.07 – -3.98)
**eGFR < 60 mL/min/1.73m^2**			
2009 CKD-EPIcr vs.			
2021 CKD-EPIcr	0.00 (0.00 – 0.00)	0.00 (0.00 – 0.00)	0.00 (0.00 – 0.00)
EKFC	0.25 (0.21 – 0.42)	-0.28 (-0.29 – -0.18)	-0.03 (-0.08 – 0.22)
**eGFR ≥ 60 mL/min/1.73m^2**			
2009 CKD-EPIcr vs.			
2021 CKD-EPIcr	-24.19 (-24.50 – -23.80)	0.05 (-0.03 – 0.15)	-24.14 (-24.52 – -23.68)
EKFC	-6.91 (-7.10 – -6.50)	0.42 (0.37 – 0.42)	-6.49 (-6.70 – -6.13)

**Procedure**			
2009 CKD-EPIcr vs.			
2021 CKD-EPIcr	-18.16 (-18.85 - –17.64)	3.35 (-3.83 – 2.73)	-21.51 (-22.38 – -20.58)
EKFC	-6.28 (-7.63 – -5.75)	2.23 (2.24 – 3.91)	-4.04 (-4.56 – -3.32)
**No procedure**			
2009 CKD-EPIcr vs.			
2021 CKD-EPIcr	1.13 (0.38 – 2.72)	-2.44 (-2.55 – -2.38)	-1.30 (-2.08 – 0.17)
EKFC	0.50 (-0.16 – 0.50)	-0.09 (-0.10 – -0.05)	0.40 (-0.22 – 0.43)
**2011–2013**			
2009 CKD-EPIcr vs.			
2021 CKD-EPIcr	0.26 (-0.06 – 1.96)	-2.33 (-2.36 – -2.20)	-2.07 (-2.32 – -0.35)
EKFC	-1.46 (-2.49 – -0.73)	0.01 (0.00 – 0.04)	-1.45 (-2.47 – -0.69)
**2014–2017**			
2009 CKD-EPIcr vs.			
2021 CKD-EPIcr	-7.65 (-9.41 – -2.91)	-3.25 (-3.28 – -2.91)	-10.91 (-12.65 – -10.14)
EKFC	-3.32 (-4.20 – -2.53)	0.01 (-0.03 – 0.04)	-3.31 (-4.15 – -2.55)
**2018–2021**			
2009 CKD-EPIcr vs.			
2021 CKD-EPIcr	-1.95 (-2.15 – -0.51)	-2.68 (-2.78 – -2.58)	-4.64 (-4.89 – -3.14)
EKFC	-1.03 (-1.73 – -0.67)	0.00 (-0.01 – 0.00)	-1.03 (-1.73 – -0.68)

AKI = acute kidney injury, NRI = net reclassification index, DM = diabetes mellitus, eGFR = estimated glomerular filtration rate
In all models, continuous eGFR variables were used.
All models were multivariable-adjusted with sex, age, hospital visit type, department, body mass index, systolic blood pressure, hemoglobin, serum sodium, procedure within 2 weeks before baseline date, history of hypertension, diabetes mellitus, and malignancy
